# Transport of Extracellular Vesicles across the Blood-Brain Barrier: Brain Pharmacokinetics and Effects of Inflammation

**DOI:** 10.3390/ijms21124407

**Published:** 2020-06-21

**Authors:** William A. Banks, Priyanka Sharma, Kristin M. Bullock, Kim M. Hansen, Nils Ludwig, Theresa L. Whiteside

**Affiliations:** 1 Geriatric Research Education and Clinical Center, Veterans Affairs Puget Sound Health Care System, Seattle, WA 98108, USA; 2 Division of Gerontology and Geriatric Medicine, Department of Medicine, University of Washington School of Medicine, Seattle, WA 98104, USA; lynckr88@gmail.com (K.M.B.); nesnah@uw.edu (K.M.H.); 3 Department of Pathology, University of Pittsburgh School of Medicine, Pittsburgh, PA 15261, USA; priyahpu@gmail.com (P.S.); ludwign@upmc.edu (N.L.); whitesidetl@upmc.edu (T.L.W.); 4 University of Pittsburgh Hillman Cancer Center, Pittsburgh, PA 15232, USA; 5 Departments of Immunology and Otolaryngology, University of Pittsburgh School of Medicine, Pittsburgh, PA 15260, USA

**Keywords:** extracellular vesicles, exosome, blood–brain barrier, pharmacokinetics, neuroinflammation, diapedesis, adsorptive transcytosis

## Abstract

Extracellular vesicles can cross the blood–brain barrier (BBB), but little is known about passage. Here, we used multiple-time regression analysis to examine the ability of 10 exosome populations derived from mouse, human, cancerous, and non-cancerous cell lines to cross the BBB. All crossed the BBB, but rates varied over 10-fold. Lipopolysaccharide (LPS), an activator of the innate immune system, enhanced uptake independently of BBB disruption for six exosomes and decreased uptake for one. Wheatgerm agglutinin (WGA) modulated transport of five exosome populations, suggesting passage by adsorptive transcytosis. Mannose 6-phosphate inhibited uptake of J774A.1, demonstrating that its BBB transporter is the mannose 6-phosphate receptor. Uptake rates, patterns, and effects of LPS or WGA were not predicted by exosome source (mouse vs. human) or cancer status of the cell lines. The cell surface proteins CD46, AVβ6, AVβ3, and ICAM-1 were variably expressed but not predictive of transport rate nor responses to LPS or WGA. A brain-to-blood efflux mechanism variably affected CNS retention and explains how CNS-derived exosomes enter blood. In summary, all exosomes tested here readily crossed the BBB, but at varying rates and by a variety of vesicular-mediated mechanisms involving specific transporters, adsorptive transcytosis, and a brain-to-blood efflux system.

## 1. Introduction

Exosomes are a subset of small extracellular vesicles (EVs) that are produced by all normal and malignant cells and are present in all body fluids [1]. EVs are heterogeneous, comprising vesicles with various sizes that originate from many different cell types. Currently, the nomenclature of these various EV types and methods for their isolation from body fluids are not firmly established [2]. Among the various EVs, exosomes are sized at 30–150 nm in diameter and are of special interest because of their origin, their ability to freely circulate and infiltrate various tissues and their molecular content mirroring that of parental cells [3]. Exosomes originate from the endosomal compartment of parent cells, where they are formed by inverse membrane invagination as intraluminal vesicles inside multivesicular bodies (MVBs) [4]. When MVBs fuse with the cell membrane, exosomes are released into the extracellular space. The orientation of surface proteins in exosome membranes resembles that in the cell membrane of parent cells [5]. These virus-sized vesicles differ from larger (250–1000 nm) microvesicles (MVs) and even larger (>1000 nm) apoptotic bodies [4]. In contrast to exosomes, MVs are formed by “budding off” from the parent cell membrane, and apoptotic bodies are products of dying cells.

Exosomes serve as an intercellular communication system, shuttling messages between cells and also conveying peptides, proteins, and genetic materials from parental to recipient cells [6]. Exosomes derived from the central nervous system (CNS) and circulating in the blood can be useful as diagnostic biomarkers and/or as markers of disease progression [7,8]. They may also signal to the peripheral immune system that a CNS injury has occurred, stimulating the trafficking of immune cells into the CNS [9]. In turn, these immune cells, reprogrammed by exosomes, can deliver substances to brain tissues, altering CNS functions [10]. For example, EVs derived from erythrocytes can cross the blood–brain barrier (BBB), contain large amounts of alpha-synuclein, and may contribute to Parkinson’s pathology [11]. As exosomes circulate freely, and appear to cross various organ barriers, attempts have been made to engineer them to deliver drugs to target tissues, including the brain. As illustrated by the work on macrophages by Batrakova et al., drug-loaded exosomes enter the brain using the same mechanisms as those employed by the parent cells [12].

Cancer cells produce an abundance of exosomes that are often enriched in angiogenic-related substances [13,14]. Exosomes derived from cancer cells can interact with the vasculature of distant tissues, including the cerebral vasculature of the brain, predisposing those tissues towards metastasis [13,14]. Cancer-derived exosomes also carry numerous immunosuppressive proteins and interfere with functions of immune cells, suppressing their anti-tumor activities in vitro and in vivo [15].

If exosomes are to serve as communication vehicles between cells in the CNS and periphery, they must negotiate the vascular blood–brain barrier (BBB). The BBB is comprised of brain endothelial cells that prevent the unregulated leakage of substances from the blood into the brain, and it further acts as an interface that regulates the exchange of substances between the brain and circulation [16,17]. Regulated transport across the BBB includes that of immune cells and viruses, and largely employs neuroimmune mechanisms [18,19,20].

While it has been reported that exosomes cross the BBB, no survey of BBB/exosome interactions have been conducted [21] and thus only limited knowledge exists about the mechanisms exosomes use to cross the BBB. Basic questions left to be answered include: Do all exosomes cross the BBB? Do exosomes from one species cross the BBB of another species? Do exosomes from cancer cells cross better or worse than those from non-cancerous cells? What is known suggests that crossing may be a common feature amongst exosomes, that exosomes could cross the BBB using a variety of mechanisms reflecting their cellular origin, and that crossing may be influenced by neuroimmune mechanisms. It appears that the mechanisms used by exosomes to cross the BBB are similar to those used by viruses, suggesting that viruses might have co-opted some of the mechanisms used by exosomes to cross the BBB [22]. In vitro work indicates that transport across the BBB involves endocytic (vesicular) processes [23].

Based on the available information, it is reasonable to hypothesize that exosomes cross the BBB primarily using transcytotic mechanisms, that is mechanisms that depend on traversing across a cell inside vesicles, such as adsorptive transcytosis [24,25]. This mechanism would be related to the pathways used to cross the BBB by immune cells, infectious agents such as viruses, some large proteins and also some nanoparticles [20,26,27,28,29,30]. Characteristics of such passage are: (I) a dependence on, and often an enhancement by, specific glycoproteins as exemplified by wheatgerm agglutinin and the HIV-1 virus; (II) mediation directly or indirectly by receptors such as mannose-6 phosphate receptor and enhancement by inflammation [20,31,32,33]. Inflammation induces alterations across the BBB of many substances by many different mechanisms, including BBB disruption and enhancement of vesicular pathways [17,29,34,35].

To address these questions, we surveyed 10 different populations of exosomes derived from culture supernatants of normal or malignant mouse or human cells. We evaluated native rates of exosome transport across the BBB, examined regional differences in brain uptake, assessed the roles of key glycoproteins in exosome transport, and determined the influence of innate immune system activation on the BBB transport of exosomes while controlling for BBB disruption.

## 2. Results

### 2.1. Exosome Characteristics

**Figure 1:**
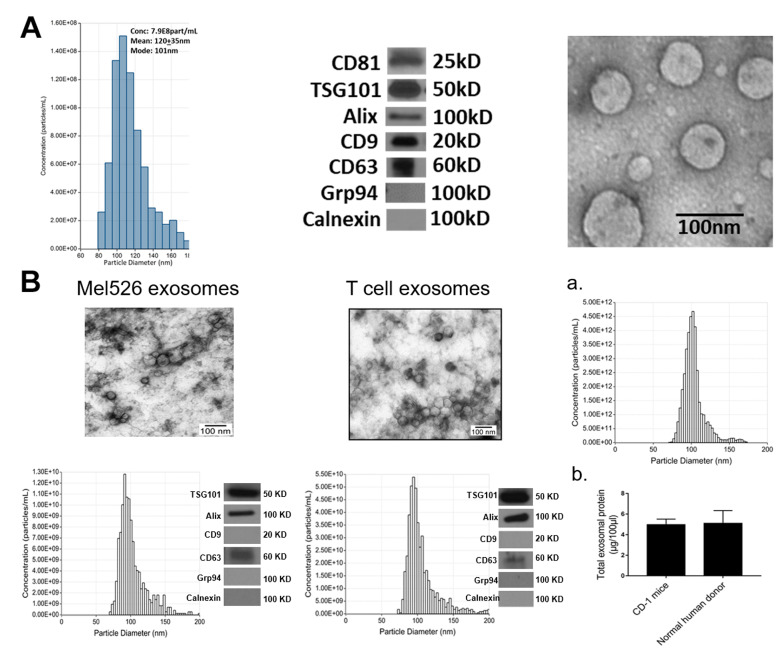
Exosome characterization: (**A**) Exosomes were isolated from supernatants of Kasumi-1, a human leukemia cell line using mini-SEC as described in Materials and Methods. Exosomes in Fraction #4 were harvested and characterized by tunable resistive pulse sensing (TRPS) to determine their diameters and concentrations (left); by Western blots to show the presence of exosome markers and absence of cytoplasmic proteins (middle); and by TEM to illustrate vesicular morphology and the size range (right). (**B**) The TEM images, size distribution profiles and Western blot profiles of Mel526 exosomes (left) and exosomes produced by human T cells (middle). (**a**) The size distribution profile of circulating exosomes in CD-1 mouse plasma; (**b**) comparisons of total exosomal protein levels of circulating exosomes in the plasma of CD-1 mice and of normal human donors. The data are means ± SD from 3 independent measurements.

EVs we refer to as “exosomes” were isolated by mini-size exclusion chromatography (SEC) from supernatants of 10 different human or murine cell lines (Table 1) and characterized according to the Minimal Information for Studies of Extracellular Vesicles 2018 (MISEV2018) criteria [2], as is routinely done in our laboratory and as we have recently described [36,37]. Briefly, exosomes were evaluated for vesicular morphology by TEM and for particle size and their diameters and numbers were determined by tunable resistive pulse sensing (TRPS) as described in Materials and Methods. Isolated exosomes were tested for the presence of various markers by Western blots. Figure 1A illustrates characteristics of exosomes isolated from supernatants of a human leukemia cell line, Kasumi-1. Figure 1B shows characteristics of exosomes produced by a human melanoma cell line, Mel526 and by human T cells. The same assessments yielding comparable profiles were performed with exosomes produced by all other cell lines. As shown in Figure 1A,B, TEM confirmed vesicular morphology of exosomes.

The TRPS results showed that the vesicle diameter ranged from 76 to 130 nm for all 10 vesicle types, with a mean diameter of 100 nm, which is consistent with our previously reported results [36]. All vesicles carried tetraspanins (CD63, CD81), although CD9 was absent from some exosomes. All exosomes were TSG101+ and Alix+, suggesting the endocytic origin, and did not carry cytoplasmic proteins, calnexin or Grp94.

The protein concentration of isolated exosomes in the SEC fractions #4 ranged between 60 and 80 µg/mL supernatant. We have also determined the numbers and diameter of circulating exosomes in plasma of normal CD-1 mice (Figure 1Ba). There were 6.7 × 10^13^) nanoparticles/mL with a mean diameter of 103 nm (SD 14.5) in mouse plasma. Interestingly, in 1 mL of normal human plasma measured in parallel, we detected a comparable number of particles (Figure 1Bb).

**Figure 2:**
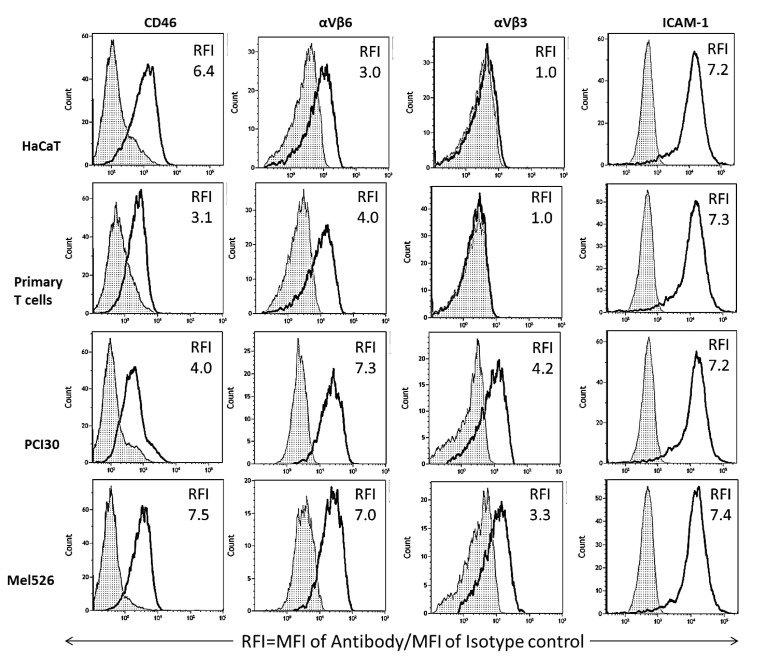
On-bead flow cytometry of exosome populations isolated from supernatants of 4 different cell lines, captured on streptavidin beads coated with biotinylated anti-CD63 mAbs and stained with the labeled detection Abs specific for CD46, *α*V*β*6, *α*V*β*3 and ICAM1 as described in Methods. Data are relative fluorescence intensity (RFI) values calculated as mean fluorescence intensity (MFI) of an experimental sample/MFI of an isotype control Ab. [White peaks = experimental samples; hatched peaks = isotope controls]. Note that exosomes produced by non-malignant cells (HaCaT and primary T cells) appear to differ from exosomes produced by malignant cells by the absence of *α*V*β*3 and lower expression levels of *α*V*β*6. Representative results are from 1 to 3 detection experiments performed with each exosome population.

Although the exosomes produced by different cell lines were all isolated by the same mini-SEC method, they represented heterogenous populations of vesicles based on their size as well as the cargo they carried. When exosomes produced by three cell lines, HaCaT, MEL526 and PCI-30, and by a primary T cell line were examined for surface expression of CD46, integrin *α*V*β*3, integrin *α*V*β*6, and ICAM1, the RFI values recorded differed for each exosome population (Figure 2). The data showed that all four exosome populations carried surface CD46, albeit at different expression levels. Normal cell-derived exosomes (HaCaT and primary T cells) did not carry *α*V*β*3 and had lower levels of *α*V*β*6 than exosomes produced by malignant cells (Figure 2). All exosomes were uniformly rich in ICAM1 (Figure 2). These data suggest that crossing of the BBB by exosomes might be in part regulated by the differences in expression levels of integrins on the exosome surface.

### 2.2. Clearance of Exosomes from Blood

**Figure 3:**
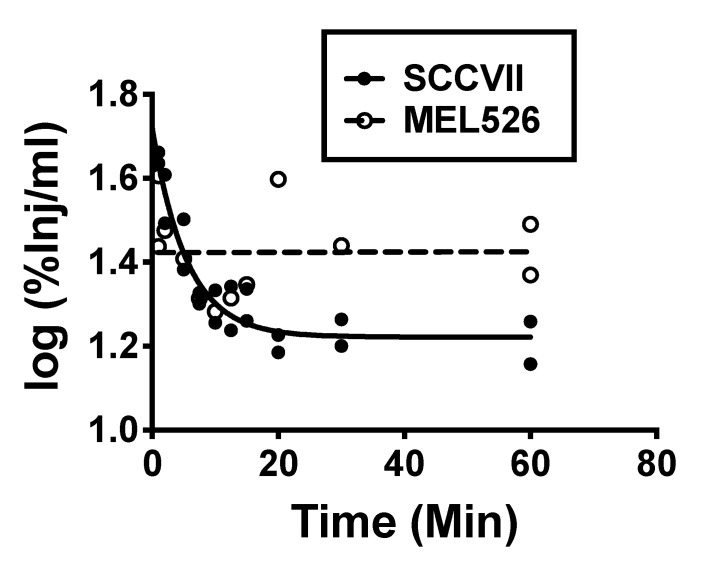
Clearance of exosomes from blood demonstrated two major patterns: phase decay, as exemplified by SCCVII (mouse tumor) exosomes and no change with time as exemplified by MEL526 (human melanoma) exosomes.

Comparison of linear and nonlinear (fitted to one phase decay: Y0 = ae^−kt^ + Plateau) for serum values expressed as log(%Inj/mL) showed two different patterns for the various exosomes. SCCVII, SCC-90, MDA-MB-231, PCI-30, and primary T cell exosomes all showed an early distribution phase followed by a plateau phase; statistical analysis showed that the preferred model was the one phase decay rather than the linear pattern. The log(%Inj/mL) vs. time curves for J774A.1, MEL526, NIH-3T3, Kasumi, and HaCaT were flat with no statistically significant variance when regressed against time. Clearance rates (*t*_½_) for the rapidly cleared group varied from 1.51 min for PCI-30 to 7.29 min for SCC-90. Figure 3 shows examples of the clearance patterns. Table 2 shows the equation parameters, estimated volumes of distribution (*Vd*) expressed in units of mL, and the half-time clearances for the early phase for those exosomes following one phase decay. Initial *Vd* was calculated by multiplying the inverse of 10*^Y0^* by 100 and the *Vd* at plateau by multiplying the inverse of 10^Plateau^ by 100. The values for *t*_1/2_ were calculated as ln2/k. For those exosome types whose blood levels did not vary with time, Table 2 lists their mean %Inj/mL and estimated *Vd*. The *Vd* was calculated as the inverse of the mean value for %Inj/mL multiplied by 100. *Vd*’s ranged from 1.41 mL for PC-I30 to a high of 5.01 mL for primary T cell exosomes with an average of about 3 mL. Our IV injections contained 0.1–1 μg of exosome protein and a distribution into 3 mL of blood would produce a blood level of 0.03–0.3 μg/mL of exosome protein. This level is about 1/150th to 1/1500th the concentration of exosome protein we found in mouse and human blood (Figure 1Ba,b. For these studies, then, we estimate that we injected about 0.13–1.3 × 10^12^ exosomes and produced blood levels of 0.45–4.5 × 10^11^ exosome particles per mL of blood.

### 2.3. Exosome Uptake by Whole Brain

**Figure 4:**
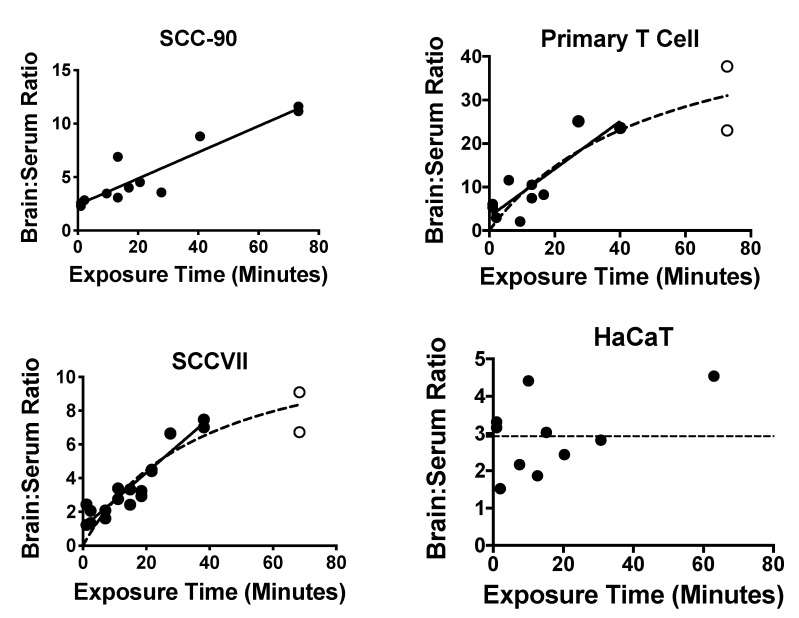
Examples of patterns for BBB passage and brain accumulation. Brain/serum ratios have been corrected for vascular space by subtracting albumin brain/serum ratios. Left upper panel illustrates continuous linear uptake of SCC-90 exosomes. Right upper and left lower panels illustrate a plateau pattern as seen with primary T cell-derived and SCCVII-derived exosomes. Only the HaCaT exosomes (right lower panel) did not show an increase in the brain/serum ratio with time. The closed circles represent data points describing the linear portion of the curve; the open circles represent data points that departed from linearity. See Table 3 for statistics.

Nine of the 10 exosome populations showed a significant correlation between their delta brain/serum ratios and exposure time, consistent with blood-to-brain passage across the BBB. Only HaCaT exosomes did not show such a correlation but did have delta brain/serum ratios (2.9 +/− 0.31, *n* = 10) that were significantly different from zero by the one sample *t*-test: *t* = 9.29, *df* = 9, *p* << 0.01. The nine exosome populations with time-dependent uptake showed two patterns: (i) linear uptake (J774A.1, SCC-90, PC130, Kasumi) throughout the study period of 60 min and (ii) a plateau in uptake (NIH-3T3, primary T cell, SCCVII, MDA-MB-231, MEL526). Figure 4 shows representative patterns and Table 3 gives pharmacokinetic details for each exosome population. The Prism program was used to determine whether the linear or hyperbolic model was the best fit. The unidirectional influx rate (*Ki*) was based on the linear portion of the brain/serum vs. exposure time relation and varied 12-fold, ranging from a low of 0.044 μL/g-min for Kasumi to a high of 0.547 μL/g-min for primary T cell.

### 2.4. Capillary Depletion

The capillary depletion method is usually used as a quality control method to determine whether a substance completely crosses the BBB (entry into parenchymal space), just binds to the luminal surface of the BEC, or is being sequestered by the BEC without subsequent entry into the brain’s interstitial fluid. Here we used both vascular washout and radioactive albumin to correct for the albumin space, as combining the two techniques produces the most reliable results. Values for uptake into the parenchymal space (Table 3) ranged from 58% to 93% with no apparent correlation with *Ki*, pharmacokinetic pattern, human vs. murine origin, or cancerous vs. non-cancerous origin of exosome populations.

### 2.5. Exosome Uptake by Different Brain Regions

**Figure 5:**
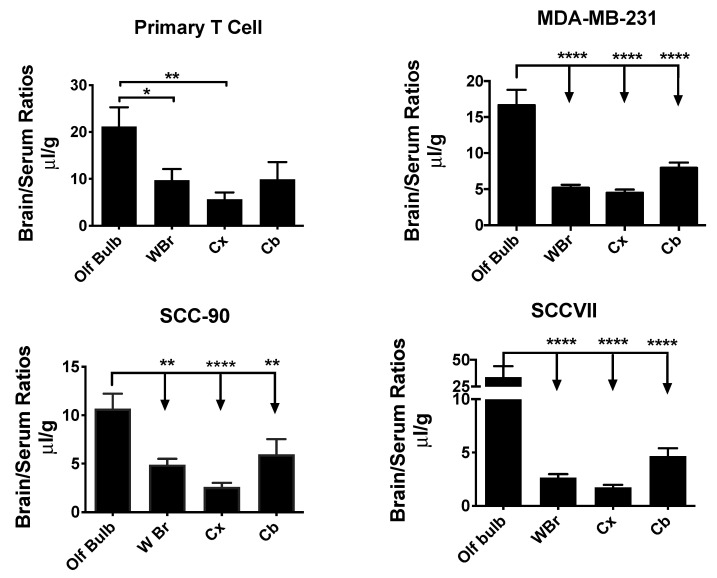
Variation in the uptake of different exosome populations by brain regions. Four exosome populations showed a significantly greater uptake by the olfactory bulb (OB) than by whole brain (WBr), cortex (Cx), or cerebellum (Cb). * *p* < 0.05, ** *p* < 0.01, **** *p* < 0.0001.

Exosome uptake by whole brain and the brain regions of olfactory bulb, cerebral cortex, and cerebellum were compared to one another by one-way ANOVA followed by the Holm–Sidak multiple comparisons test. Four exosome types showed differences among brain regions and these are shown in Figure 5. The olfactory bulb (OB) showed more uptake than any of the other brain regions for SCC-90 [F(3,25) = 10.9, *p* < 0.001], SCCVII [F(3,30) = 11.9, *p*< 0.001], and MDA-MB-231 [F(3,37) = 22.9, *p* < 0.001]. For primary T cell exosomes, the OB had a significantly higher uptake than the WBr or CX but not the CB [F(3,33) = 4.88, *p* < 0.01]. The other six exosomes showed no statistical differences in uptake by various brain regions.

### 2.6. Effects of LPS on Exosome Uptake

**Figure 6:**
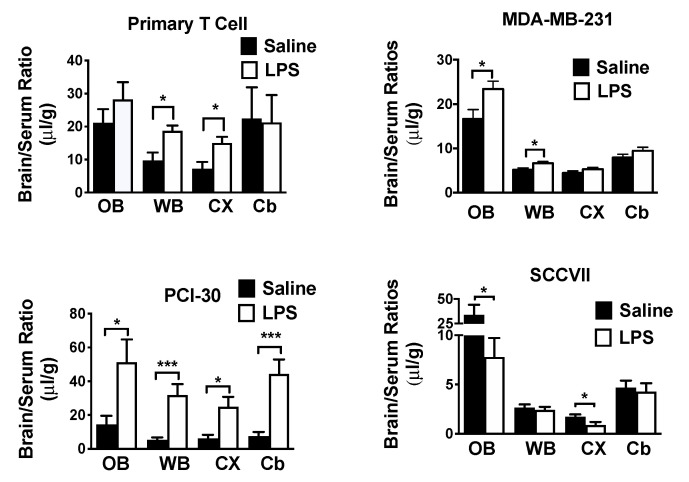
Effects of LPS on exosome uptake by various brain regions. Upper left: uptake of primary T cell-derived exosomes by whole brain (WBr) and cortex (Cx), but not by OB (olfactory bulb) or cerebellum (Cb), was increased by LPS. Upper right: for MDA-MB-231-derived exosomes; LPS increased uptake by the OB and the WBr. Lower left: for PCI-30-derived exosomes, LPS increased uptake into all the assessed regions. Lower right: for SCCVII-derived exosomes, LPS decreased uptake by the OB and the CX. * *p* < 0.05, *** *p* < 0.001.

LPSwas used to determine the effects of inflammation/neuroinflammation on exosome uptake. LPS had no significant effect on the volume of distribution or clearance rate from blood for any exosome population (data not shown). The effects of LPS on exosome uptake by WBr, OB, CX, and CB were compared by *t*-test. LPS had an effect on seven of the exosome populations, increasing the uptake of 6/7 human-derived exosome populations and decreasing uptake of one of the three mouse populations (Table 4). Figure 6 shows 4 of the exosome populations whose regional uptake was affected by LPS. For primary T cell exosomes, the WBr (t = 2.86, df = 15, *p* < 0.05) and the CX (t = 2.65, df = 15. *p* < 0.05) had statistically significant increases (Figure 6, upper left panel). The OB (t = 2.43, df = df = 18, *p* < 0.05) and the WBr (t = 2.54, df = 18, *p* < 0.05) increased for MDA-MB-231 (Figure 5, upper right panel). For PCI-30, the OB (t = 2.25, df = 12, *p* < 0.05), WBr (t = 4.04, df = 16, *p* < 0.001), CX (t = 2.70, 18, *p* < 0.05) and CB (t = 4.13, df = 16, *p* < 0.001) all increased to a statistically significant level (Figure 5, lower left panel). SCCVII exosomes (Figure 5, lower right panel) represented the only exosome type for which values decreased with LPS treatment, significantly so for the OB (t = 2.31, df = 11, *p* < 0.05) and CX (t = 2.18, df = 16, *p* < 0.05). For HaCaT exosomes, LPS significantly increased values for the CX (t = 2.21, df = 17, *p* < 0.05) from 1.26 +/− 0.4 (*n* = 9) to 3.19 +/− 0.74 (*n* = 10). Uptake by the CB increased from 6.49 +/− 1.05 (*n* = 8) to 11.5 +/− 1.52 (*n* = 6) for Kasumi exosomes (t = 2.81, df = 12, *p* < 0.05). For SCC-90 exosomes, there were significant increases for WBr (4.09 +/− 0.61, *n* = 5 vs. 8.10 +/− 0.30, *n* = 6, df = 5.0, *p* < 0.001) and for CX (2.61 +/− 0.41, *n* = 10 vs. 4.19 +/− 0.31, *n* = 8, t = 2.97, df = 19, *p* < 0.01). LPS had no effect on brain uptake for MEL526, NIH-3T3, or J774A.1 exosomes.

### 2.7. Effects of WGA and M6P on Exosome Uptake

**Figure 7:**
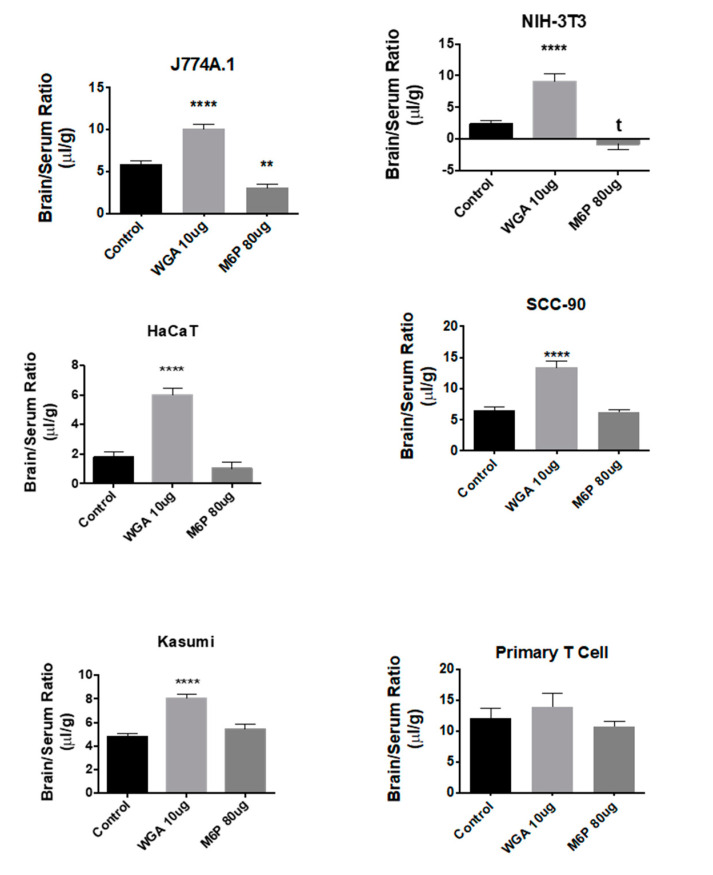
Effects of WGA and M6P on exosome uptake by whole brain. Shown are data for 5/10 tested exosome populations in which WGA significantly enhanced uptake. M6P inhibited uptake of 1/10 exosome populations tested (see J774A.1). Neither WGA nor M6P had an effect on primary T cell-derived exosomes, ** *p* < 0.01, **** *p* < 0.001.

The effects of WGA and M6P on exosome uptake by the brain were compared by one-way ANOVA followed by Tukey’s multiple comparison test. For all groups, the *n* ranged between 8 and 10 per statistical cell. WGA robustly increased brain uptake of three of the four exosome populations derived from non-cancer sources [J774A.1: F(2,25) = 45.5, *p* << 0.01; NIH-3T3: F(2,23) = 29.5, *p* << 0.01; HaCaT: F(2,27) = 47.7, *p* << 0.01] and two of the six derived from cancer cells [SCC-90: F(2,27) = 34.1, *p* << 0.01; Kasumi: (F(2,21) = 28.2, *p* << 0.01] (Figure 7). There was no apparent relation between WGA’s effects and origin (species or cancerous state) of the exosome population nor the response to LPS (Table 4). M6P had only one statistically significant effect and that was to lower uptake for J774A.1, although there was also a statistical trend for NIH-3T3. For comparison, the effect of WGA and M6P on primary T cell-derived exosomes is also shown in Figure 7 for which whole brain uptake of exosomes was not affected by either WGA or M6P.

### 2.8. Brain-to-Blood Efflux of Exosomes

**Figure 8:**
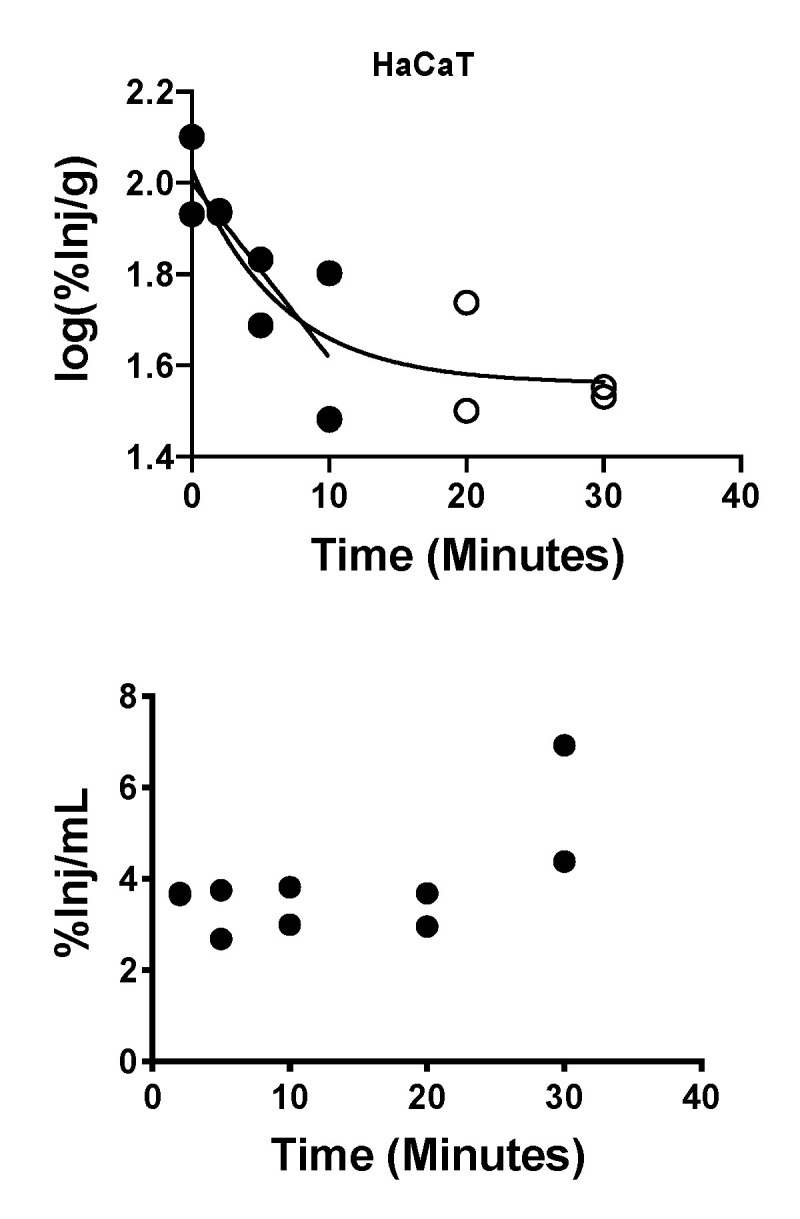
Brain-to-blood efflux of HaCaT-derived exosomes after ICV administration. *Upper panel*: open circles show that exosome clearance from brain had a half-life of 7.85 min; open circles show that exosomes reach an equilibrium after about 10 min. *Lower panel*: Appearance of HaCaT exosomes in blood was stable at a mean of 3.9%.

The ability of HaCaT exosomes to cross in the brain-to-blood direction was tested with the intracerebroventricular (ICV) injection method [38,39]. Results showed a very rapid clearance rate with a plateau after 10 min. Nonlinear one phase decay was a better fit than a linear curve for all data, but the first 10 min fitted to a linear model showed a half-time disappearance of 7.85 min (Figure 8, upper panel). The %T was fitted to a hyperbolic model and had a theoretical maximum (B max) of 72% (data not shown). Radioactivity appearing in blood after the ICV injection of I-HaCaT exosomes confirmed brain-to-blood transfer and was stable (that is, no correlation between blood levels and time) at a mean value of 3.9% (Figure 8, lower panel).

## 3. Discussion

Exosomes represent a universal intercellular communication system [6]. The ability of exosomes to deliver messages to or from the CNS likely depends on exosome access to and crossing of the vascular blood–brain barrier (BBB). Exosome–brain interactions are of special interest, because of the implication of exosome-mediated mechanisms in human diseases such as Alzheimer’s disease, Parkinson’s disease, and metastatic brain cancers [7,11,13,14,40]. Limited data are available that document in vivo exosome interactions with the vascular BBB, and this study is the first to examine how exosomes derived from several cancerous and non-cancerous cell types cross the BBB. Another objective of the study was to compare the BBB transport rates of human-derived and mouse-derived exosomes injected into mice. We and others have reported that human tumor-derived exosomes injected IV into mice are not rejected or inactivated as might be expected, but rather effectively promote tumor growth, metastasis, angiogenesis, or immune suppression [14]. The selected cell lines used in this study for exosome production and listed in Table 1 reflect these objectives.

Crossing the BBB first requires that the exosome be present in the blood and, therefore, understanding their clearance from blood is critical. We found that exosome populations produced by various human or mouse tumor cells, non-malignant HaCaT cells and by primary human T cells segregated into two broad categories: those that were rapidly cleared from circulation with half-lives ranging from 1.51 to 7.29 min and those that were not rapidly cleared from the vascular space over the time course of this study. These clearance patterns did not segregate into human vs. mouse or cancer vs. non-cancer categories. Either clearance pattern eventually settled into a steady state; that is, a prolonged time period where the %Inj/mL value did not vary. In almost all cases, the %Inj/mL for the steady state was less than 50%, which means the volume of distribution exceeded the vascular space of the mouse, indicating uptake by peripheral tissues. Previous work has found that exosomes are rapidly cleared from blood and that blood levels are determined by a balance between secretion and clearance rates [41,42,43]. The presence of a prolonged steady state indicates that a third factor influencing blood levels is involved, that of exchange between the circulation and the sequestering peripheral tissues.

Using multiple-time regression analysis, we found that all the exosomes we tested crossed the BBB. However, the unidirectional influx rate varied over ten-fold from a low of 0.044 µL/g-min for Kasumi to a high of 0.524 µL/g-min for primary T cell exosomes. It should be noted that these and all brain uptake measures were corrected for vascular space (and any BBB leakage which might have occurred from other treatments) by measuring values for the simultaneously injected albumin. Albumin is the largest and most reliable of all markers typically used for measuring vascular space and is the best suited for measuring disruption when studying larger substances, such as proteins and exosomes, whereas Evans blue and other such dyes are the least useful [44].

We found that some exosome populations showed blood-to-brain uptake patterns that were hyperbolic rather than linear. One explanation for a hyperbolic pattern is that equilibrium between brain and blood is being influenced by the presence of an efflux (that is, a brain-to-blood) transport system. From this perspective, HaCaT exosomes represent a special case. The brain/serum values for HaCaT exosomes were much greater than the brain’s vascular space as measured by albumin, demonstrating its ability to cross the BBB. Yet the absence of a linear relation between brain/serum ratios and the exposure time suggests an extremely potent efflux system. Using the ICV method, we demonstrated a robust efflux system for HaCaT-derived exosomes. An efflux system would explain why CNS-derived exosomes are found in the circulation [7], raising the question of their role in disease states and supporting their usefulness as biomarkers of CNS diseases. Our results indicate that efflux may vary amongst exosomes and, therefore, not all CNS-derived exosomes will be proportionately represented in the blood.

Uptake mechanisms for transport across the BBB can vary across brain regions, as illustrated by the transporter for tumor necrosis factor [45], or can be homogenous, as illustrated by glucose transport [46]. We observed a rather homogenous uptake pattern among the exosome populations. Only four exosome populations showed statistically significant variations among the uptake rates of brain regions, and only the olfactory bulb was different from other brain regions. As these included exosomes from human non-cancer, human cancer, and mouse cancer cell lines, neither species nor cancer state seemed to be relevant to this uptake. Only for HaCaT exosomes, the olfactory bulb failed to show an arithmetic increase in uptake. This suggests that the binding/transport mechanisms for exosomes tend to be higher in the olfactory bulb, but otherwise tend to be evenly distributed throughout the brain.

To further characterize the BBB transport, we tested the effects of LPS, WGA, and M6P on exosome transport across the BBB. LPS, WGA, and M6P influence BBB penetration through different mechanisms. LPS affects the passage across the BBB of many substances and cells and does so through various mechanisms. As examples, LPS enhances immune cell trafficking through upregulation of selectins, HIV-1 free virus through cytokine release and enhanced adsorptive transcytosis, insulin transport via nitric oxide dependent mechanisms, and albumin entry by disrupting the BBB via prostaglandin-dependent pathways [34,47,48,49]. WGA enhances adsorptive transcytosis of glycoproteins that contain sialic acid and *N*-acetyl-d-glucosamine [50]. M6P blocks substances crossing the BBB that use the M6P receptor [51].

In our study, LPS increased uptake of six different exosome populations by some brain regions and decreased uptake for one exosome type. Interestingly, LPS did not increase uptake of exosomes from the macrophage cell line J774A.1. LPS stimulates trafficking of immune cells across the BBB including macrophages. Batrakova et al. have previously shown that exosomes derived from macrophages have increased uptake after LPS treatment [38]. Here, LPS treatment increased uptake by cortex for five exosome populations, by whole brain for four, by olfactory bulb for three, and by cerebellum for two. However, uptake by one region was not predictive of uptake by another region or by the whole brain, nor did it segregate as to species, cancer condition, or effect of WGA. Given the many mechanisms by which LPS can alter BBB permeability, it is not surprising that here LPS resulted in a complex pattern of enhanced exosome uptake. Again, we ruled out that these results with LPS were caused by BBB disruption by having corrected all results with co-injected radioactive albumin.

WGA stimulated uptake by whole brain for 5 exosome types. This suggests that sialic acid or *N*-acetyl-d-glucosamine is likely part of the glycoproteins that these exosomes target. By binding to these glycoproteins, WGA stimulates the adsorptive transcystosis of other molecules that bind to these same molecules [20,24,25,32]. Thus, while stimulation of BBB transport by WGA argues that five exosomes (J774A.1, NIH-3T3, HaCAT, SCC-90, Kasumi) bind to brain endothelial cell glycoproteins containing sialic acid or *N*-acetyl-d-glucosamine, the results also suggest that the other five exosomes (primary T cell, SCVII, MEL526, MDA-MB-231, PCI-30) do not bind to them. M6P blocked uptake of one exosome population produced by NIH-3T3 cells, demonstrating dependence on the mannose 6-phosphate receptor for transport of this exosome [51].

Two options for exosomes taken up by the BBB are either to cross the endothelial cell barrier completely to enter the brain or to be sequestered within the brain endothelial cells. Sequestration could allow exosomes to exert their effects on brain endothelial functions [14], whereas complete passage would facilitate effects on the brain. Using the capillary depletion method, we determined that the degree to which each exosome population completely crossed the BBB varied from 58% to 92% (Table 3). Transcytosis is a time-dependent event, so one would expect there to be some exosomes present in the capillary at any given time. But this wide range suggests that some exosome populations may be partially sequestered by capillaries or that there is considerable variation in their rates of transcytosis. How and whether this variation is related to the integrins differentially carried by exosomes, as our and other studies suggest, deserves further attention.

It has been recently suggested that CD46 is the major BBB-associated receptor used by exosomes derived from metastatic melanoma cells for crossing the BBB [26]. We observed that exosomes derived from normal or malignant cells carried high levels of CD46, supporting the role of CD46 as a major BBB receptor for exosomes crossing the BBB. Erythrocyte-derived EVs were reported to use mechanisms akin to adsorptive transcytosis for crossing the BBB [7]. While most previous studies investigated receptors that might be present on brain endothelial cells, we used on-bead flow cytometry to detect the presence of these receptors on the exosome surface. We selected primary T cells as they produced the most rapidly transported exosome populations, HaCaT as the slowest, and PCI-30 and MEL526 as malignant cells with intermediate transport rates. We found CD46 and ICAM-1 on all exosome populations, showing that receptors typically thought to be on brain endothelial cells are also carried by exosomes. Alpha V beta 3 was the only glycoprotein not expressed on every exosome population examined. With the exception of ICAM-1, which was highly and uniformly expressed on all four exosome populations, the other glycoproteins were variably expressed. No single protein correlated with rates or patterns of uptake, consistent with exosomes using a variety of glycoprotein receptors to cross the BBB.

One might speculate that mouse exosome populations would cross the mouse BBB more readily than human exosome populations or that being of cancerous lineage would alter transport in comparison to being non-cancerous. However, neither of these factors exerted any consistent effect on BBB transport rates. Table 4 shows that none of the mouse lines responded to LPS, whereas six of seven human lines did. Otherwise, neither species of origin nor cancer status affected transport rate, patterns of uptake, or responses to LPS or WGA. This finding, the diversity of responses to WGA and LPS, and the lack of cell surface proteins to be predictive of transport rates and responses demonstrate that the initiators and regulators of exosome transcytosis are highly individualistic for a given exosome.

Overall, our results show that exosomes cross the BBB, but do so at varying rates and by a variety of mechanisms. Some exosomes show a preference for uptake by the olfactory bulb, but otherwise exosomes tend to have a homogeneous uptake within the CNS. Some exosomes respond to LPS and WGA, but response to one of these substances is not predictive of its response to the other. Nonlinearity of brain uptake is common among exosomes and may indicate efflux (brain-to-blood transport). Uptake rate, linearity vs. nonlinearity of uptake, preference for the olfactory bulb, and response to either LPS or WGA are not predicted by species of origin or whether the exosome is derived from a cancerous or noncancerous cell line. The M6P receptor is implicated in the transport of NIH-3T3-derived exosomes. These patterns suggest that whereas uptake by brain of circulating exosomes is likely near universal, the mechanisms of uptake are diverse and may include at the very least specific transporters, various initiators of adsorptive transcytosis and other vesicular mechanisms, and are influenced by brain-to-blood efflux.

## 4. Materials and Methods

### 4.1. Cell Lines

Human melanoma cell line, MEL526; human HPV(+) head and neck squamous cell carcinoma (HNSCC) cell line, UM-SCC-90; human breast cancer cell line, MDA-MB-231; human leukemic cell line, Kasumi; and human keratinocytes, HaCaT, were grown in RPMI media supplemented with 10% exosome-depleted fetal bovine serum (FBS). FBS was ultracentrifuged at 100,000× *g* overnight and filtered using a 022 µm filter (Millipore). The same lot of FBS was used for culture of all the cell lines listed in Table 1, except for human T cells (see below). Human HPV (−), HNSCC cell line, PCI-30; mouse squamous cell carcinoma, SCCVII; mouse macrophage cell line, J774A.1; and mouse fibroblast, NIH-3T3, were grown in DMEM supplemented with 10% exosome depleted FBS. Cells were cultured to confluence under growth conditions optimized for each cell line as previously described [36]. Supernatants were harvested and processed for exosome isolation [36]. Cell lines were monitored for mycoplasma and all were found to be negative.

### 4.2. Culture of Primary Human T Cells

Peripheral blood was obtained from healthy volunteers who signed a consent form approved by the Institutional Review Board (IRB no. 0403105) at the University of Pittsburgh. Venous blood was collected in heparin tubes and was processed immediately for recovery of peripheral blood mononuclear cells (PBMC) by centrifugation on Ficoll-Paque Plus gradients (GE Healthcare Bioscience, Pittsburgh, PA, USA). The recovered cells were washed in RPMI medium (Lonza, Basel, CH) supplemented with 10% (*v*/*v*) exosome-depleted FBS (Gibco, Thermo Scientific, Pittsburgh, PA, USA) and immediately used for experiments. T cells were isolated by negative selection on AutoMACS (Miltenyi Biotec, San Diego, CA, USA) using an isolation kit from Miltenyi Biotec as previously described by us [52]. T cells were activated using CD3/CD28 T cell activator (25 μL/mL, Stemcell, Vancouver, BC, CA) and IL-2 (150 IU/mL, PeproTech, Bionity, Rocky Hill, CT, USA) in freshly-prepared RPMI for 48–72 h prior to culture for exosome production.

### 4.3. Isolation of Exosomes by Mini-Size Exclusion Chromatography

Preparation of cell supernatants for exosome isolation was previously described [36]. Briefly, aliquots (50 mL) of each supernatant were centrifuged at 2000× *g* for 10 min at room temperature (RT) and then at 10,000× *g* for 30 min at 4 °C. Each supernatant was filtrated using a 0.22 µm bacterial filter (Millipore) and then concentrated to 1 mL using Vivacell 100 concentrators (MWCO 10,000 kDa Sartorius Corp, Bohemia, NY, USA) at 2000× *g*. Aliquots of concentrated supernatants (1 mL) were loaded on mini-SEC columns and eluted with phosphate buffered saline (PBS) and collected as 1 mL fractions. Exosomes eluted in fraction #4 were characterized as previously described [53].

### 4.4. BCA Protein Assay

The protein concentration of exosomes in fraction #4 was determined using BCA protein assay kit (Pierce Biotechnology, Rockford, IL, IDA) as per manufacturer’s instructions. The eluted #4 fractions were concentrated on Vivaspin (Sartorius) 500 concentrators (MWCO 100,000) to a desired concentration of 1 mg/mL for animal studies.

### 4.5. Transmission Electron Microscopy

Transmission electron microscopy was performed at the Center for Biologic Imaging at the University of Pittsburgh. Freshly-isolated exosomes (fraction #4) were placed on a copper grid coated with 0.125% Formvar in chloroform. After the addition of 50 µL of uranyl-acetate solution, exosomes were visualized in a Hitachi H-7100 transmission electron microscope (TEM; Hitachi High Technologies, Tokyo, Japan), as described previously [53].

### 4.6. Tunable Resistive Pulse Sensing (TRPS)

The concentration and size distribution of the particles in fraction #4 were analyzed with TRPS (qNano, Izon Science Ltd, Cambridge, MA, USA) using a NP100 nanopore at a 45.05 mm stretch, a voltage of 0.64 V and 17 mbar pressure. Samples were calibrated with 114 nm carboxylated polystyrene beads at a concentration of 2 × 10^11^ particles/mL. Data recording and analysis were performed using the Izon software (version 3.2).

### 4.7. Western Blot Analysis of Exosomes

To concentrate exosomes isolated by mini-SEC (fraction #4) to 0.5 mL, 100K Amicon Ultra centrifugal filters (EMD Millipore) were used for centrifugation at 4000× *g*. Vesicles were lysed and were separated on SDS/PAGE gels. Proteins were transferred onto a PDVF Immobilon-P membrane (EMD Millipore) for Western blot analysis. Each lane was loaded with 5 μg of protein from fraction #4, and PVDF membranes were incubated overnight at 4 °C with antibodies specific for TSG101 (1:1000, ab30871, Abcam); Alix (1:1000, ab2171S, Cell Signaling); CD9 (1:500, ab65230, Abcam); CD63 (1:500, ab59479, Abcam); CD81 (1:500, abPA5-13582, ThermoFisher); calnexin (1:2000, ab2433S, Cell Signaling); and Grp94 (1:1000, ab2104, Cell Signaling).

### 4.8. On-Bead Flow Cytometry for Exosomes

The on-bead flow cytometry method for exosomes was previously validated by western blots as described by us (see supplementary data in [54]). To quantify expression of CD46, integrins and ICAM-1 on the surface of exosomes isolated from cell line supernatants, exosomes were first captured on ExoCap™ Streptavidin magnetic beads (supplied as the ExoCap^TM^ streptavidin kit) from MBL International (Woburn, MA) as described by us [55]. Briefly, exosomes (10 μg/100 uL PBS) were co-incubated with biotin-labeled anti-CD63 mAb (clone H5C6; Biolegend, San Diego, CA) adjusted to the concentration of 1 ug for 2 h at RT. Next, a 100 μL aliquot of beads was added to each tube, and the tubes were again incubated for 2 h at RT. Samples were washed once with PBS using a magnet. The bead/anti-CD63Ab/exosome complexes were then co-incubated with the labeled detection Abs. Next, the complexes were washed twice with PBS using a magnet and were resuspended in 300 uL of PBS for antigen detection by flow cytometry. The detection method provided the RFI (relative fluorescence intensity) values calculated as the ratio of MFI with detection antibody/MFI with isotype control antibody. The following detection antibodies were used: anti-CD46 mAb (clone TRA-2-10) purchased from Biolegend)); anti-Integrin αVβ3 (clone 23C6) from eBioscience (San Diego, CA), anti-CD54 (HA58) was from Invitrogen (San Diego, CA) and Integrin αVβ6 was from Abcam (MA, USA).

### 4.9. Radioactive Labeling and Purifying

Exosomes isolated by SEC from supernatants of various tumor cell lines and non-malignant cell lines (see Table 1) were characterized according to the criteria suggested by MISEV2018 [2], as described by us previously [36,37]. The vesicles were radioactively labeled with 0.5mCi ^125^I (Perkin Elmer, Waltman, MA, USA) using the chloramine-T method and purified on an Illustra NAP-5 (GE Healthcare, Piscataway, NJ, USA) column eluting with PBS. All exosomes showed greater than 80% acid precipitation after labeling and studies were conducted the day of labeling. Bovine serum albumin (Sigma, St. Louis, MO, USA) was labeled with ^131^I using the chloramine-T method or ^99m^ Tc (GE Healthcare) using the stannous tartrate method. Both I-Alb and Tc-Alb were purified on a column of G-10 Sephadex (GE Healthcare).

### 4.10. Circulating Levels of Exosomes in Mice

Eight-week-old CD-1 male mice (Charles River) were maintained in a 12/12-h light dark cycle and given ad lib water and food. All studies were performed under protocols approved by the local animal use committee and at an Association for Assessment and Accreditation Laboratory Animal Care International (AAALAC) facility. To determine levels of autologous exosomes circulating in a normal mouse, we sacrificed 6 mice and pooled plasma to obtain a quantity sufficient for exosome isolation by mini-SEC. Plasma was centrifuged for 10 min at 2000× *g* and then for 30 min at 10,000× *g* and filtered using a 0.22 µm filter. Next, 1 mL of plasma was placed on a mini SEC column, exosomes were eluted with PBS and collected as fraction #4 as previously reported for the isolation of exosomes from human plasma [56].

### 4.11. Intravenous (IV) Time Curve and Brain Uptake

Mice were anesthetized by giving an intraperitoneal (IP) injection of 0.15–0.2 mL 40% urethane. The left jugular vein was exposed and an injection of 0.2 mL lactated Ringer’s solution containing 1 × 10^6^ cpm of a radioactively-labeled exosomes and 1 × 10^6^ cpm of radioactively-labeled albumin was delivered into the left jugular vein. At time points between 1 and 60 min, blood was collected from the carotid artery. Blood was centrifuged at 5400× *g* for 10 min and 50 µL serum was collected. The brain was removed and weighed. In some studies, where indicated, the olfactory bulb (OB) (not included in whole brain), cortex (CX), and cerebellum (CB) were separately counted and weighed. For these studies, whole brain (WBr) values were determined by summing total levels of radioactivity and total weights (minus OB). Brain, brain regions, and serum were placed into a gamma counter and the levels of radioactivity were measured. Results for serum were expressed as the percent of the injected dose per mL of blood (%Inj/mL). Results for brain and brain regions were expressed as the tissue/serum ratio in units of μL/g. For each individual tissue, its ratio for radioactive albumin was subtracted from its ratio for the radioactive exosome, yielding the “delta” value which reflected extravascular uptake.

### 4.12. Lipopolysaccharide (LPS) Preparation and Treatment

Mice were given an IP injection of 0.2 mL of 0.9% NaCl solution with half the injections containing 3 mg/kg LPS (Salmonella enterica serotype typhimurium, L6511, Sigma, St. Louis, MO, USA). Mice received 3 such injections at times (t) = 0, 6, and 24 h. At 28 h after the first injection, mice were anesthetized with urethane and studied as outlined above, except that time points after the radioactive injection ranged from 1–20 min. The WBr did not include the OB.

### 4.13. Effects of Mannose 6-Phosphate and Wheatgerm Agglutinin on Tissue Uptake of Exosomes

Mice were given an IP injection of 0.15–0.2 mL 40% urethane. The left jugular vein and the right carotid artery were exposed. An injection of 0.2 mL lactated Ringer’s solution containing 1 × 10^6^ cpm of the radioactive exosome and 1 × 10^6^ albumin was given into the jugular vein at t = 0. In some mice, this injection contained either 10 ug wheatgerm agglutinin (WGA; Sigma) or 80 ug mannose 6-phosphate (M6P; Sigma). At t = 20 min, carotid artery blood and brain were collected and the results expressed as above.

### 4.14. Capillary Depletion with Washout

Mice were anesthetized, the jugular vein exposed, and 0.2 mL of lactated Ringers solution containing 1 × 10^6^ cpm of a radioactive exosome and 1 × 10^6^ of radioactive albumin was injected into the jugular vein. After 20 min, blood was collected from the abdominal aorta. The thorax was then immediately opened, the descending thoracic aorta clamped, and both jugular veins were severed. Twenty milliliters of cold lactated Ringer’s solution was perfused over 1 min into the left ventricle of the heart, thus washing out the vascular space of the brain. The WBr was removed and homogenized (10 strokes) in an ice-cold glass homogenizer containing 0.8 mL of physiological buffer (10 mM HEPES, 141 mM NaCl, 4 mM KCl, 2.8 mM CaCl_2_, 1 mM MgSO_4_, 1 mM NaH_2_PO_4_, and 10 mM d-glucose, pH 7.4). Dextran solution (26%) was added to the homogenate and homogenized a second time (three strokes). Using a swinging bucket rotor, the homogenate was centrifuged at 5400× *g* for 15 min at 4 °C. The pellet (brain microvessels) and supernatant fractions (brain parenchyma) were separated and levels or radioactivity were measured in each fraction in a gamma counter. Results were expressed as the percentage of total radioactivity found in the parenchymal fraction (%Par).

### 4.15. Intracerebroventricular (ICV) Injection of Exosomes

An ICV method was used to assess the brain-to-blood efflux rate for HaCaT exosomes [38,39]. Mice were anesthetized with an IP injection of 0.15 mL of 40% urethane (Sigma-Aldrich, St. Louis, MO). The scalp was removed, and a hole was made in the skull (0.5 mm posterior and 1 mm lateral to the bregma) and 1 μL of lactated Ringers solution containing 2.5 × 10^4^ CPM of I-HaCaT exosomes was injected into the lateral ventricle of the brain using a 1.0 μL Hamilton syringe. At 2, 5, 10, 20, or 30 min after this ICV injection, blood was obtained from the carotid artery and the mice were decapitated. The WBr was removed and weighed. The amount of I-HaCaT exosomes available for transport at t = 0 was estimated in mice that had been overdosed with urethane and had been dead for 10–20 min before receiving the I-HaCaT exosomes [23]. Levels of radioactivity in brains and arterial serum were determined in a gamma counter. Two sets of 3 mice were studied at each time point and the log of the median value for each set was plotted against time. Linear and nonlinear regressions lines for brain data were fitted and compared with the statistical software of Prism. Linear regression was fitted for the arterial serum data. The percentage of the available I-HaCaT exosomes that was transported out of the brain (%T) was determined with the equation:

%T = [100(cpm/g)_0_ − (cpm/g)_10_]/(cpm/g)_0_ (1)

where (cpm/g)_0_ is the mean level of cpm/g for the t = 0 group and (cpm/g)_10_ is the individual mouse’s level of cpm/g at t = 10 min.

### 4.16. Statistical Analysis

To calculate clearance from blood, the %Inj/mL values were transformed to log values and regressed against time. We compared linear and nonlinear (one phase decay) models using the Prism statistical package (GraphPad Inc, San Diego, CA, USA) and reported the best fit. Mice were studied over a 60 min period. Blood-to-tissue unidirectional influx rates (*Ki*’s; units of μL/g-min) were calculated by regressing the delta values (tissue/serum ratios corrected for the albumin space) against exposure time (Expt) with multiple-time regression analysis [57,58]. This method yields the *Ki* and also the initial volume of distribution in the tissue (Vi; units of μL/g). Each regression is reported with its *n*, *r*^2^, and *p* value. Means are reported with their standard error terms and *n*’s. Study period was 60 min. For regional brain uptake rates and effect of lipopolysaccharide (LPS) on brain uptake, the study time was too short to produce statistically significant correlations between brain/serum ratios and Expt., so values were combined irrespective of time. For comparison of brain region uptake, a one-way analysis of variance (ANOVA) followed by the Holm–Sidak multiple comparisons test was performed. For LPS, *t*-tests were performed. Means are reported with their standard errors and *n*-group.

## Figures and Tables

**Table 1:** Sources of Exosomes and Characteristics of Producer Cell Lines ^a^.

Designation	Species	Tissue	Non-Cancerous/Cancer
J774A.1	Mouse	Macrophage	Non-Cancerous
NIH-3T3	Mouse	Fibroblast	Non-Cancerous
Primary T Cell	Human	T Cell	Non-Cancerous
HaCaT	Human	Keratinocyte	Non-Cancerous
SCCVII	Mouse	Oral Squamous	Cancer
MEL526	Human	Melanoma	Cancer
MDA-MB-231	Human	Breast	Cancer
PCI-30	Human	Head & Neck	Cancer
SCC-90	Human	Head & Neck	Cancer
Kasumi	Human	Leukemia	Cancer

^a^ Exosomes were isolated by size exclusion chromatography (SEC) from supernatants of the above listed cell lines as described in Methods.

**Table 2:** Serum Pharmacokinetics and Estimated Volumes of Distribution ^a^.

Exosome	*Y0* (*Vd*)	Plateau (*Vd*)	*k* (*t*_1/2_)	R^2^ (*n*)
Primary T Cell^NC^	1.30 (5.01)	0.989 (10.2)	0.227(3.05)	0.610 (12)
SCC-90^C^	1.63 (2.34)	1.32 (4.79)	0.095 (7.29)	0.538 (13)
SCCVII^C^	1.72 (1.91)	1.22 (6.03)	0.182 (3.80)	0.899 (20)
MDA-MB-231^C^	1.77 (1.70)	1.39 (4.07)	0.339 (2.04)	0.542 (12)
PCI-30^C^	1.81 (1.41)	1.41 (3.89)	0.459 (1.51)	0.464 (13)
	%Inj/mL: Mean +/−SE (*Vd*)			*n*
Kasumi^C^	34.8 +/− 2.68 (2.87)			13
HaCaT^NC^	43.2 +/− 1.48 (2.31)			11
NIH-3T3^NC^	45.2 +/− 3.05 (2.21)			12
J774A.1^NC^	33.6 +/− 2.19 (2.98)			13
MEL526^C^	27.3 +/− 2.02 (3.66)			12

^a^ Values for *Vd* calculated as outlined in Results with units of mL. t_1/2_ calculated as discussed in Results with units of min. Superscript: NC = noncancerous; superscript C = cancerous.

**Table 3:** Pharmacokinetics for Whole Brain Uptake.

Exosome	*Ki*	*Vi*	*n* *	*r*^2^ *	*p*< *	Pattern	%Par
J774A.1^NC^	0.083 ± 0.017	1.72	12	0.710	0.001	Linear	92 ± 0.6
NIH-3T3^NC^	0.103 ± 0.040	1.11	10	0.455	0.05	Plateau	58 ± 3.7
Primary T Cell^NC^	0.547 ± 0.116	3.19	10	0.736	0.005	Plateau	85 ± 2.9
HaCaT^NC^						Flat	58 ± 8.1
SCC-90^C^	0.123 ± 0.015	2.40	12	0.866	0.001	Linear	77 ± 2.0
PCI-30^C^	0.057 ± 0.011	4.11	13	0.721	0.001	Linear	71 ± 2.9
SCCVII^C^	0.162 ± 0.015	1.07	18	0.884	0.001	Plateau	93 ± 3.0
MDA-MB-231^C^	0.098 ± 0.034	3.66	10	0.514	0.05	Plateau	64 ± 4.5
MEL526^C^	0.091 ± 0.035	1.7	7	0.579	0.05	Plateau	72 ± 3.9
Kasumi^C^	0.044 ± 0.013	3.3	11	0.568	0.01	Linear	79 ± 4.6

*Ki* in units of μL/g-min reported with SE; *Vi* in units of μL/g; %Par is the percent in parenchyma (capillary depletion), reported as mean with its standard error for an *n* = 3; * for linear portion of curve only. HaCaT did not show a statistically significant correlation between brain/serum ratios and time and its mean brain/serum ratio was 2.9 +/− 0.31 (*n* = 10). Superscript NC = noncancerous; superscript C = cancerous.

**Table 4:** Correlations among responses to WGA and LPS effects on brain uptake as a function of cell species and cancerous/noncancerous states.

	LPS No Increase	LPS Increase
WGA No Increase	SCCVII *	**Primary T Cell**
	**MEL526**	**MDA-MB-231**
		**PCI-30**
WGA Increase	**J774A.1**	**HaCaT**
	**NIH-3T3**	**SCC-90** **Kasumi**

Green: mouse non-cancerous;Blue: human non-cancerous;Red: mouse cancerous;Purple: human cancerous; * LPS induced a decrease in uptake.

## Abbreviations

BBB	Blood–brain barrier
CNS	Central nervous system
EVs	Extracellular vesicles
LPS	Lipopolysaccharide
MVs	Microvesicles
MVBs	Multivesicular bodies
t_1/2_	Half-time clearance
WGA	Wheatgerm agglutinin
%Inj/mL	Percent of the injected dose present in one ml of serum
